# Effects of rutin on acrylamide-induced neurotoxicity

**DOI:** 10.1186/2008-2231-22-27

**Published:** 2014-02-13

**Authors:** Vahideh Sadat Motamedshariaty, Sara Amel Farzad, Marjan Nassiri-Asl, Hossein Hosseinzadeh

**Affiliations:** 1 Pharmaceutical Research Center, School of Pharmacy, Mashhad University of Medical Sciences, Mashhad, Iran; 2 Cellular and Molecular Research Centre, Department of Pharmacology, School of Medicine, Qazvin University of Medical Sciences, Qazvin, Iran; 3 Pharmacodynamics and Toxicological Department, Pharmaceutical Research Center, School of Pharmacy, Mashhad University of Medical Sciences, Mashhad, Iran

**Keywords:** Rutin, Acrylamide, MTT assay, Neural toxicity, Antioxidant

## Abstract

**Background:**

Rutin is an important flavonoid that is consumed in the daily diet. The cytoprotective effects of rutin, including antioxidative, and neuroprotective have been shown in several studies. Neurotoxic effects of acrylamide (ACR) have been established in humans and animals. In this study, the protective effects of rutin in prevention and treatment of neural toxicity of ACR were studied.

**Results:**

Rutin significantly reduced cell death induced by ACR (5.46 mM) in time- and dose-dependent manners. Rutin treatment decreased the ACR-induced cytotoxicity significantly in comparison to control (P <0.01, P < 0.001). Rutin (100 and 200 mg/kg) could prevent decrease of body weight in rats. In combination treatments with rutin (50, 100 and 200 mg/kg), vitamin E (200 mg/kg) and ACR, gait abnormalities significantly decreased in a dose-dependent manner (P < 0.01 and P < 0.001). The level of malondialdehyde significantly decreased in the brain tissue of rats in both preventive and therapeutic groups that received rutin (100 and 200 mg/kg).

**Conclusion:**

It seems that rutin could be effective in reducing neurotoxicity and the neuroprotective effect of it might be mediated via antioxidant activity.

## Introduction

Risk factors in food are either of chemical or microbiological origin, or a combination of both. Acrylamide (ACR), one such risk factor, is a possible human carcinogen [1]. It is an industrial chemical and has been known as an occupational hazard for decades [2,3]. However, in recent years, ACR has been found to form in fried and baked starchy foods during cooking [4,5]. This latter finding has greatly raised public concerns over ACR’s potential health risk due to dietary exposure to people. ACR is a neurotoxic chemical and can cause peripheral and central neuropathy in humans and laboratory animals [6].

Early morphological studies suggested that both human and experimental neurotoxicities were mediated by cerebellar Purkinje cell injury and by degeneration of distal axons in the peripheral (PNS) and central nervous system (CNS) [7]. In addition to neurotoxicity, considerable experimental data from rodent studies has shown that ACR induces reproductive toxicity (e.g. reduced litter size) and genotoxic effects (e.g. DNA strand breaks, dominant lethal mutation) [3,8,9]. There are several reports that antioxidant agents could rescue neurotoxicity induced by ACR via increasing antioxidant activity [10-14].

Rutin (3, 3′, 4′, 5, 7 -pentahydroxyflavone-3-rhamnoglucoside) is a flavonoid of the flavonol type that is found in many typical plants, such as buckwheat, passion flower, apple and tea. It is also an important dietary constituent of foods and plant-based beverages [15]. Rutin has several pharmacological properties, including antioxidant, anticarcinogenic, cytoprotective, vasoprotective, cardioprotective and neuroprotective activities [16-23]. In humans, it attaches to the iron ion (Fe), preventing it from binding to hydrogen peroxide, which would otherwise create a highly reactive free radical that may damage cells [24].

The present study was therefore designed to investigate the protective effects of rutin in prevention and treatment of neural toxicity induced by ACR.

## Materials and methods

### Materials

RPMI 1640 and FBS were purchased from Gibco. (4,5-dimethylthiazol-2-yl)-2, 5-diphenyl tetrazolium (MTT), rutin hydrate ≥94% (HPLC), TBA (2-thiobarbituric acid), n-butanol, potassium chloride, phosphoric acid and ACR were obtained from Merck. Vitamin E was purchased in injectable form from Osveh Company, Iran.

### Cell culture

PC12 cells were obtained from Pasteur Institute (Tehran, Iran). Cells were maintained at 37°C in a humidified atmosphere (90%) containing 5% CO_2_. Cells were grown in RPMI 1640 medium supplemented with 10% (v/v) heat-inactivated foetal bovine serum, 100 U/ml penicillin and 100 μg /ml streptomycin.

### Cell viability

The viability of cultured cells was determined by assaying the reduction of 3-(4,5-dimethyl thiazol-2-yl)-2,5- diphenyl tetrazolium bromide (MTT) to formazan [25]. Briefly, PC12 cells were cultured in a 96-well microliter plate at a density of 5000 cell/well. After pretreatment with rutin (0.5, 1, 1.5, 2.5, 5, 10, 20, 40, 80 and 160 μM/ml) for 24 h, ACR at concentration of 5.46 mM was added to each well. The cells were then incubated for 48 h and then treated with MTT solution (0.5 mg/ml PBS) for 1 h at 37°C. Upper mediate replaced with dimethyl sulfoxide (DMSO). The absorbance was measured at 570 nm (630 nm as reference) in a plate reader (TECAN infinit M200) [14].

### Experimental animals

Male Wistar rats (200–270 g) were housed in colony rooms with 12/12 h light/dark cycle at 21 ± 2°C and had free access to food and water. All animal experiments were carried out in accordance with Mashhad University of Medical Sciences, Ethical committee Acts.

### Experimental design

To induce neurotoxicity in rats, the animals were exposed to ACR at a daily dose of 50 mg/kg intraperitoneally (i.p.) [26]. All doses of rutin were selected as our previous work [18]. This daily dose and the corresponding route have been well characterized with respect to neuropathological expression and neurological deficits. For our study on the preventive effect, the rats were divided at random into 7 groups (n = 6 in each group) and treatment was given as follows:

1) Saline (negative control) for 14 days

2) ACR (50 mg/kg, i.p.) for 14 days

3) Rutin (50 mg/kg, i.p.) for 3 days alone and afterward rutin (50 mg/kg, i.p.) + ACR (50 mg/kg, i.p.) for 11 days

4) Rutin (100 mg/kg, i.p.) for 3 days alone and afterward rutin (100 mg/kg, i.p.) + ACR (50 mg/kg, i.p.) for 11 days

5) Rutin (200 mg/kg, i.p.) for 3 days alone and afterward rutin (200 mg/kg, i.p.) + ACR (50 mg/kg, i.p.) for 11 days

6) Rutin (200 mg/kg, i.p.) for 14 days

7) Vitamin E (200 mg/kg, i.p.) (positive control) for 3 days alone and afterward vitamin E (200 mg/kg, i.p.) + ACR (50 mg/kg, i.p.) for 11 days

Behavioural testing to assess preventive effects was performed on the 15th day [14].

After studying doses of rutin in preventing neurotoxicity, the most effective dose was used to assess its therapeutic effect. For our study, the rats were divided at random into 5 groups (n = 6 in each group) and treatment was given as follows:

1) Normal saline (negative control) for 14 days

2) ACR (50 mg/kg, i.p.) for 3 days alone and afterward ACR (50 mg/kg, i.p.) + rutin (200 mg/kg, i.p.) for 11 days

3) ACR (50 mg/kg, i.p.) for 3 days alone and afterward ACR (50 mg/kg, i.p.) + vitamin E (200 mg/kg, i.p.) for 11 days

4) ACR (50 mg/kg i.p.) for 7 days alone and afterward ACR (50 mg/kg, i.p.) + rutin (200 mg/kg i.p.) for 7 days

5) ACR (50 mg/kg i.p.) for 7 days alone and afterward ACR (50 mg/kg, i.p.) + vitamin E (200 mg/kg, i.p.) for 7 days

Behavioural testing was performed on the 15th day [14].

### The behavioural index (gait scores) examination

After completion of treatment, the rats were placed in a clear plexiglass box and were observed for 3 min. Following observation, a gait score was assigned from 1 to 4, where 1 = a normal, unaffected gait; 2 = a slightly affected gait (foot splay, slight hind limb weakness and spread); 3 = a moderately affected gait (foot splay, moderate hind limb weakness, moderate limb spread during ambulation) and 4 = a severely affected gait (foot splay, severe hind limb weakness, dragging hind limbs, inability to rear) [27].

### Biochemical assay

Furthermore, a test was conducted to determine lipid peroxidation by determining thiobarbituric acid reactive products (TBAR) of lipid peroxidation in brain tissue of rats [28,29]. Malondialdehyde (MDA) is the final product of lipid peroxidation and its complex with TBA is red and has maximum absorbance at 532 nm; MDA was assessed as the lipid peroxidation indicator. Briefly, the brain tissue was homogenized in cold KCl solution (1.5%) to obtain a homogenous suspension (10%). To a 10-ml tube, 0.5 ml of suspension was poured and 3 ml phosphoric acid (1%) and 1 ml TBA were added, following which the tube was placed in a boiling water bath for 45 min. Then, the suspension was cooled, 4 ml n-butanol added and the tube placed in vortex for complex mixing (1 min). Following this, the suspension was centrifuged (20000 rpm speed, 20 minutes) to separate the red-coloured upper phase and its absorbance was determined using the spectrophotometer. The standard curve for MDA (0–40 μM) was prepared [30].

### Statistical analysis

Results are expressed as mean ± SEM. For in vitro assay, IC_50_ values were calculated using the method described by Litchfield and Wilcoxon method (PHARM/PCS software version 4). Statistical analyses were performed with ANOVA followed by the Tukey–Kramer test to compare the differences between means. Differences were considered statistically significant when P < 0.05.

## Results

### Effect of ACR in PC12 cells

**Figure 1:**
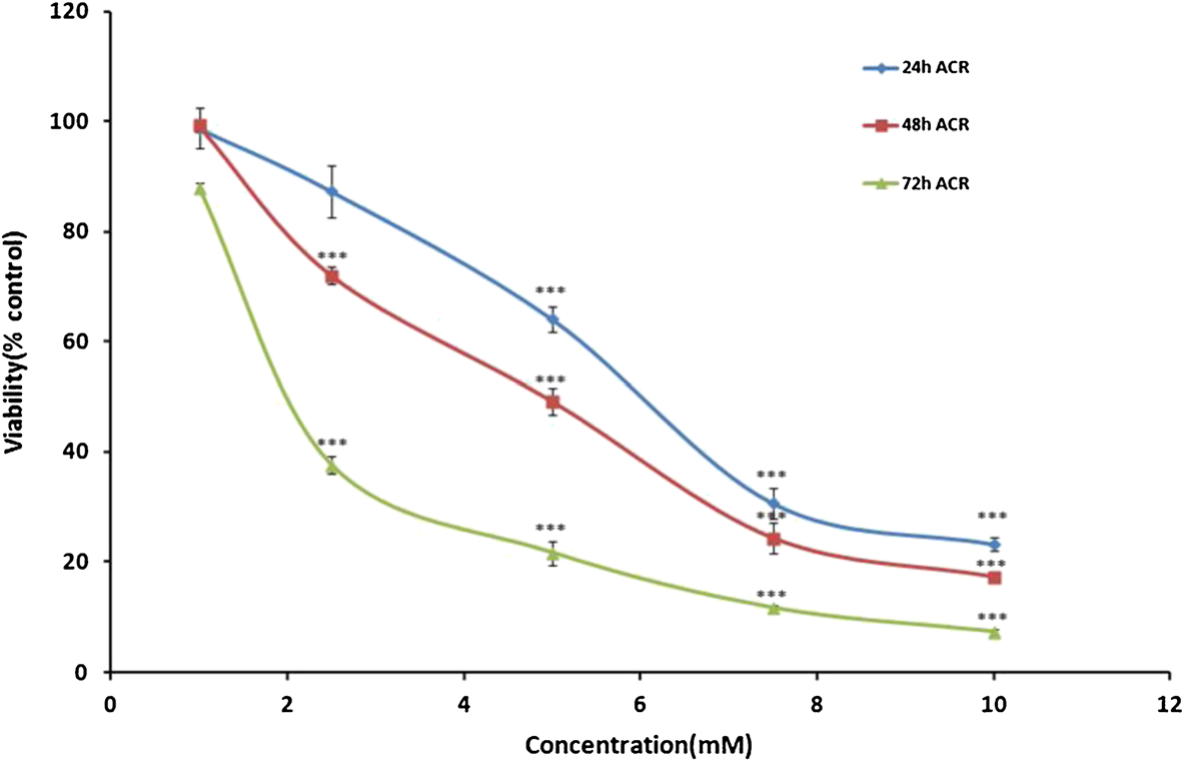
**Cell viability of PC12 cells after exposure to different concentrations of acrylamide (ACR) for 24, 48 and 72 h.** Cell viability was determined by MTT test. Data are expressed as mean ± SEM of 6 separate experiments. ACR was solubilised in PBS. For control cells PBS was used. ***P < 0.001 vs. control cells.

The PC12 cells were treated with different concentration of ACR for 24, 48 and 72 h. Following this, the cell viability was measured using MTT test. Treatment of cells with ACR decreased viability in time- and dose-dependent manners, as shown in Figure 1. The IC_50_ (50% inhibitory concentration) value for treatment of PC12 cells with ACR for 48 h was 5.46 mM.

### Effect of rutin on ACR-induced cytotoxicity in PC12 cells

**Figure 2:**
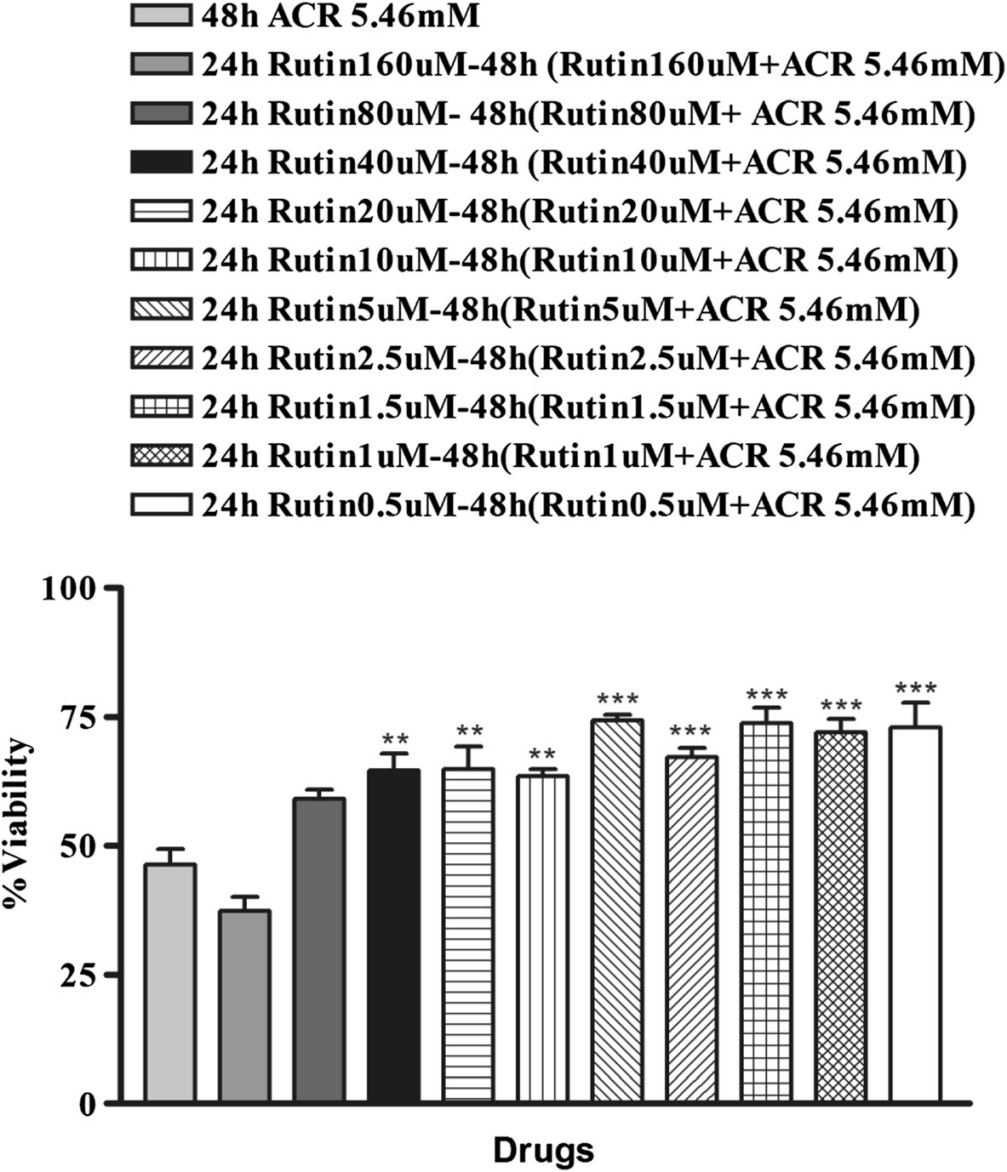
**Effect of rutin on ACR-induced cytotoxicity in PC12 cells.** Cells were pretreated with different concentration of rutin (0.5, 1, 1.5, 2.5, 5, 10, 20, 40, 80 and 160 μM/ml) for 24 h and ACR (5.46 mM) was added to each well later, followed by evaluation of cell viability using MTT assay. Data are expressed as the mean ± SEM of 6 separate experiments (n = 6). **P < 0.01, ***P < 0.001 vs. ACR-treated cells.

PC12 cells were treated with different concentrations of rutin (0.5, 1, 1.5, 2.5, 5, 10, 20, 40, 80 and 160 μM/ml) for 24 h and then ACR (5.46 mM) was added to each well, followed by evaluation of cell viability using MTT assay. Rutin treatment decreased the ACR-induced cytotoxicity significantly in comparison to control (Figure 2).

### Effect of ACR on rat body weight and the protective effect of rutin

**Figure 3:**
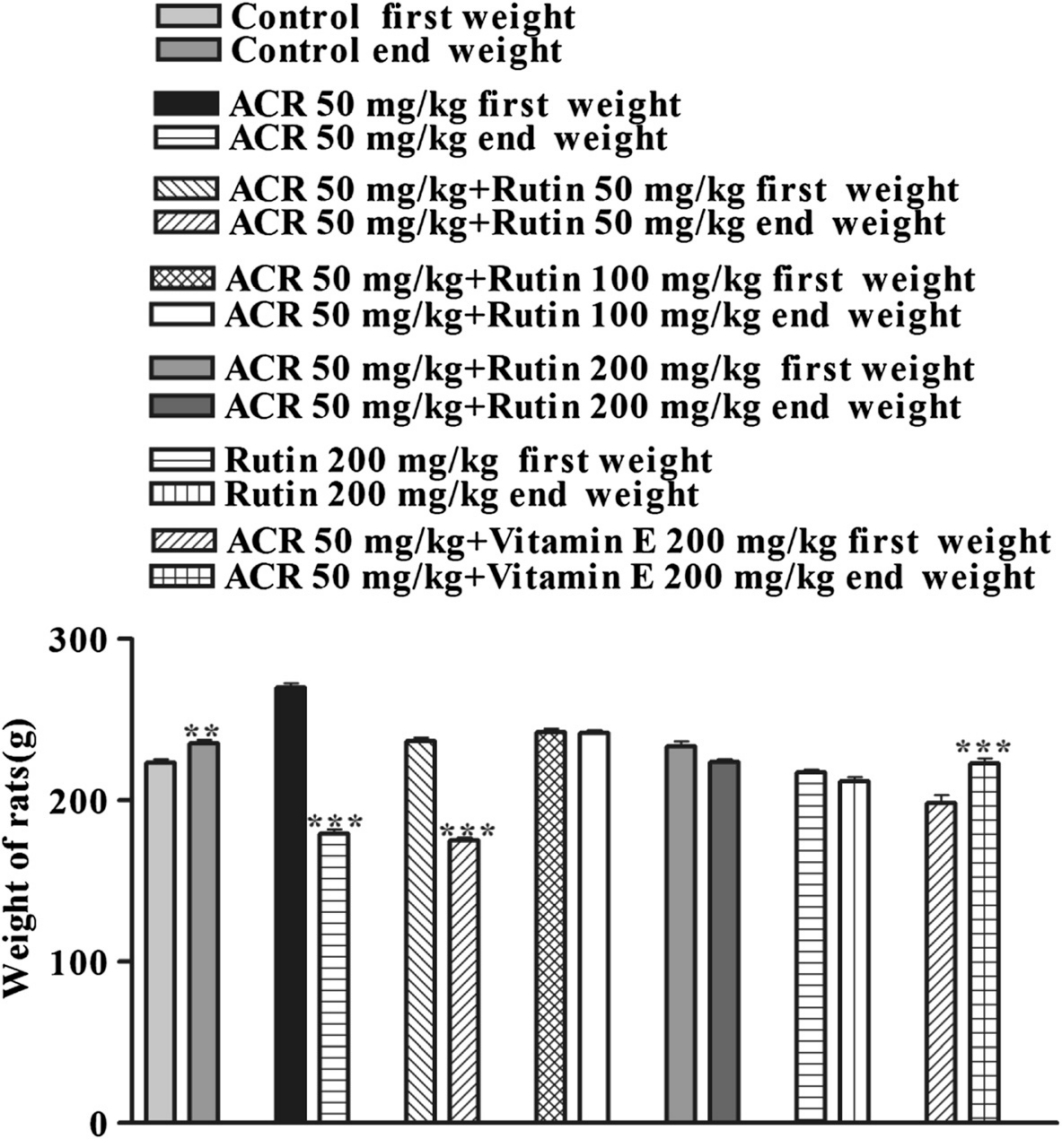
**Protective effect of rutin (after 14 days of ACR exposure) by comparison between the initial and end weight of rats.** Data are expressed as mean ± SEM, (n = 6), **P < 0.01, ***P < 0.001 vs. first weight of each group treated with drugs.

The body weight of the ACR group decreased 33.6% after 14-day exposure to ACR (Figure 3). Treatment with rutin at a dose of 50 mg/kg failed to prevent weight loss caused by ACR. Therefore, body weight decreased after 14 days. However, rutin at doses of 100 and 200 mg/kg prevented decrease of body weight in rats. Vitamin E (200 mg/kg) increased the body weight in rats (P < 0.001) (Figure 3).

### Effect of ACR on rat body weight and the therapeutic effect of rutin

**Figure 4:**
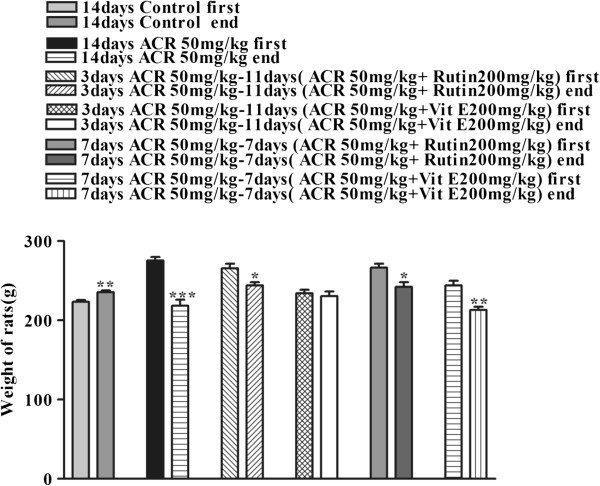
**Therapeutic effect of rutin (after 14 days of ACR exposure) by comparison between the initial and end weight of rats.** Data are expressed as mean ± SEM, (n = 6). *P < 0.05, **P < 0.01, ***P < 0.001 vs. first weight of each group treated with drugs.

In the control group, only the body weight increased after 14 days compared with the first weight (P < 0.01). In other groups, the body weight significantly decreased compared with the first weight. However, in the group administered vitamin E, there was no significant change in body weight after 3 days (Figure 4).

### Effect of ACR on the behavioural index (gait scores) in rats and the protective effect of rutin

**Figure 5:**
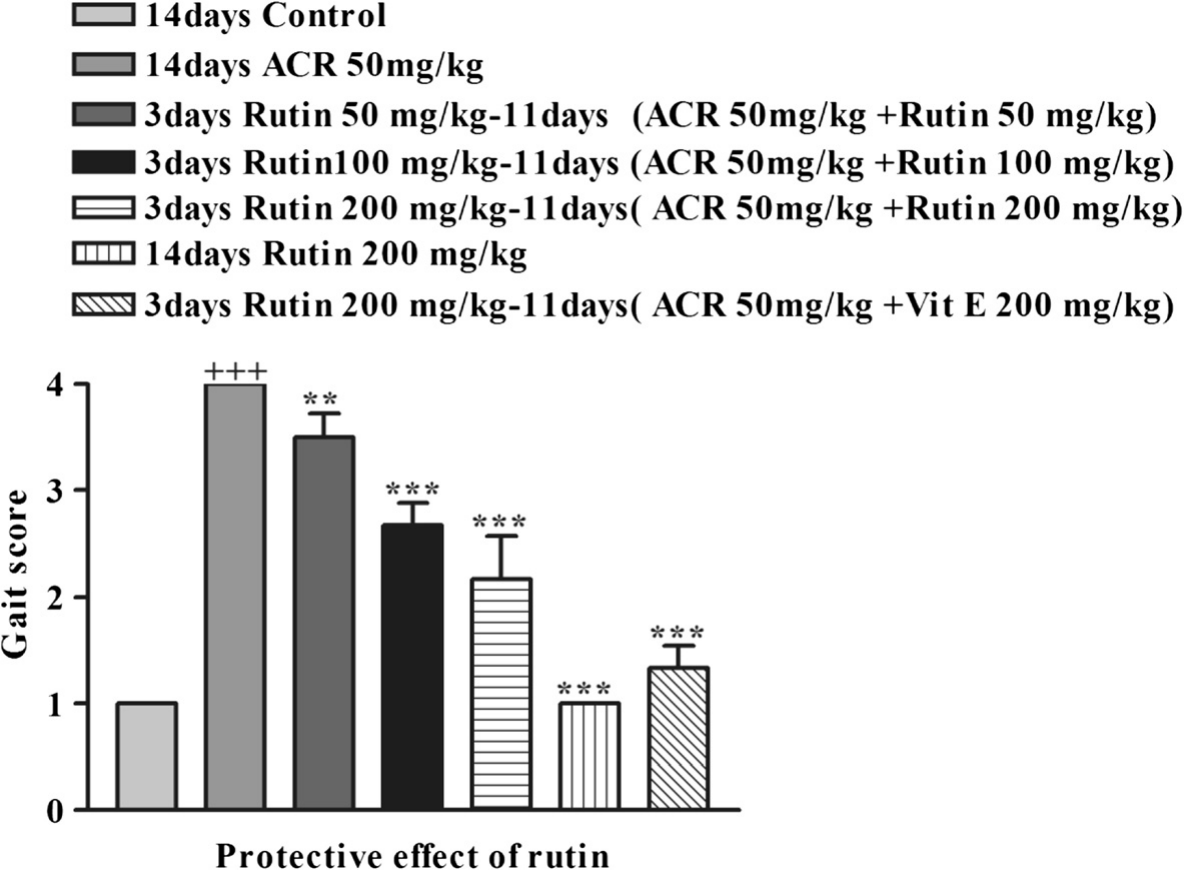
**Protective effect of rutin on behavioural index (gait scores) in rats during treatment with ACR (50 mg/ kg, i.p.) for 14 days.** Data are expressed as the mean ± SEM, (n = 6). +++P < 0.001 vs. control, *P < 0.05 **P < 0.01, ***P < 0.001 vs. ACR-treated animals.

Exposure to ACR (50 mg/kg, i.p.) for 14 days led to progressive gait abnormalities in rats, as shown in Figure 5. In groups of rats that were treated with combination of rutin (50, 100 and 200 mg/kg), vitamin E (200 mg/kg) and ACR, gait abnormalities significantly decreased in a dose-dependent manner (P < 0.01 and P < 0.001 vs. ACR-treated group) (Figure 5).

### Effect of ACR on the behavioural index (gait scores) in rats and the therapeutic effect of rutin

**Figure 6:**
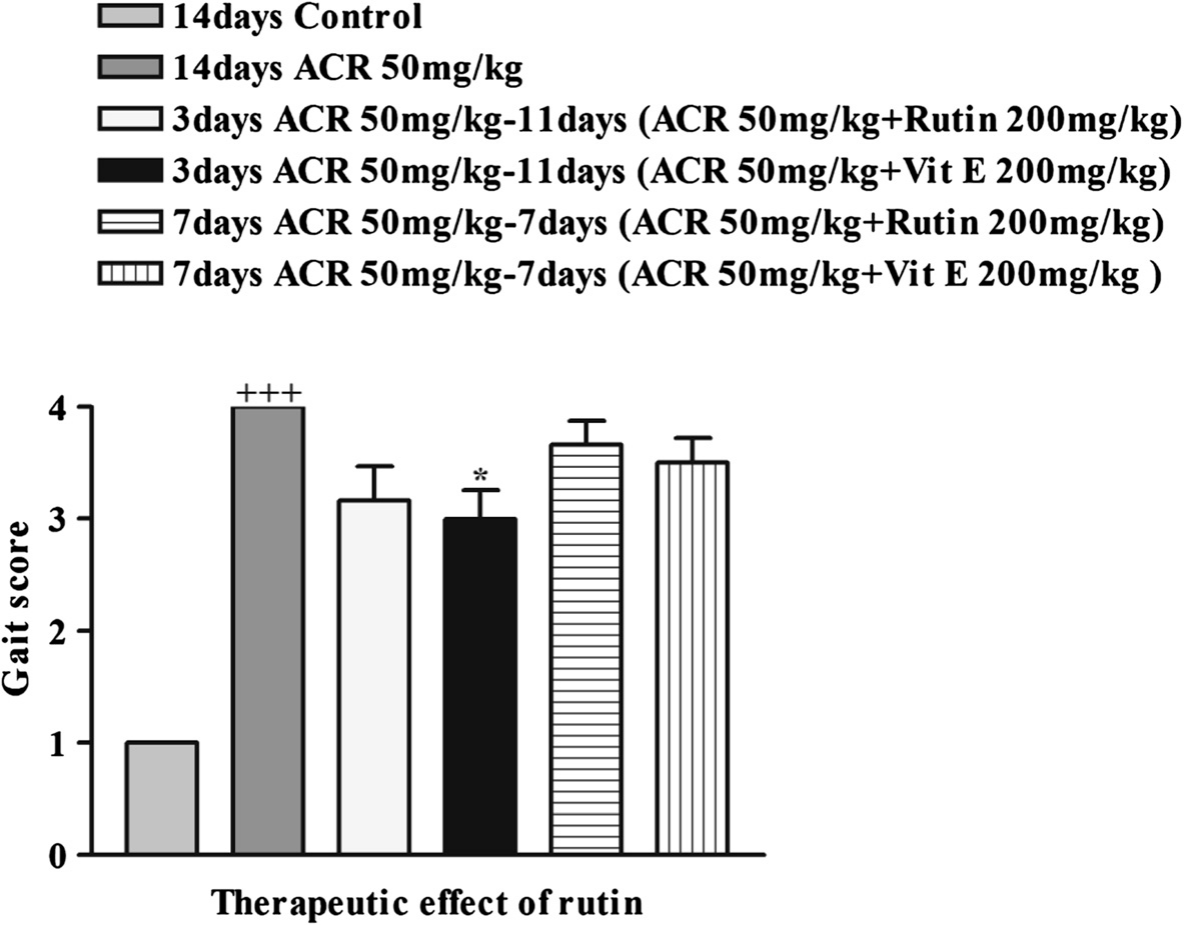
**Therapeutic effect of rutin on behavioural index (gait scores) in rats during treatment with ACR (50 mg/ kg, i.p.) for 14 days.** Data are expressed as the mean ± SEM, (n = 6). +++P < 0.001 vs. control, *P < 0.05 vs. ACR-treated animals.

The groups in which the therapeutic effect of rutin were evaluated, ACR was administered to the animals for 3 and 7 days and then treated 11 and 7 days with rutin and vitamin E with combination of ACR, respectively. In all groups, gait abnormalities decreased but the effect of vitamin E was only significant compared with ACR after 3 days treatment (P < 0.05) (Figure 6).

### Effect of rutin in biochemical assay

There was an increase in the MDA levels following ACR administration compared with the control group (P < 0.001). The MDA levels significantly reduced dose dependently in all groups that were administrated rutin for preventive and therapeutic purposes (except on administration of 50 mg/kg rutin) (Tables 1 and 2).

**Table 1:** Protective effect of rutin on lipid peroxidation following ACR administration

**Groups**	**MDA concentration (nmol/g) tissue**
14 days Control	45.75 ± 3.92
14 days ACR 50 mg/kg	92.58 ± 3.69^+++^
14 days Rutin 200 mg/kg	75.52 ± 2.55***
3 days Rutin 50 mg/kg-11 days (ACR 50 mg/kg + Rutin 50 mg/kg)	69.39 ± 4.26
3 days Rutin 100 mg/kg-11 days (ACR 50 mg/kg + Rutin 100 mg/kg)	49.7 ± 7.77*
3 days Rutin 200 mg/kg-11 days (ACR 50 mg/kg + Rutin 200 mg/kg)	47.76 ± 3.05***
3 days Vit E 200 mg/kg-11 days (ACR 50 mg/kg + Vit E 200 mg/kg)	52.67 ± 4.38***

Values are mean ± SEM (n = 6). ^+++^P < 0.001 vs. control, *P < 0.05, ***P < 0.001 vs. ACR-treated animals.

**Table 2:** Therapeutic effect of rutin on lipid peroxidation following ACR administration

**Groups**	**MDA concentration (nmol/g) tissue**
14 days Control	45.75 ± 3.92
14 days ACR 50 mg/kg	92.58 ± 3.69^+++^
3 days ACR 50 mg/kg-11 days (ACR 50 mg/kg + Rutin 200 mg/kg)	65.10 ± 5.03**
3 days ACR 50 mg/kg-11 days (ACR 50 mg/kg + Vit E 200 mg/kg)	60.75 ± 3.46***
7 days ACR 50 mg/kg-7 days (ACR 50 mg/kg + Rurin 200 mg/kg)	69.67 ± 5.630*
7 days ACR 50 mg/kg-7 days (ACR 50 mg/kg + Vit E 200 mg/kg)	68.87 ± 5.14*

Values are mean ± SEM (n = 6). ^+++^P < 0.001 vs. control, *P < 0.05, **P < 0.01, ***P < 0.001 vs. ACR-treated animals.

## Discussion

In this study, the neuroprotective effect of rutin on ACR-induced cytotoxicity in PC12 cells and ACR-induced neurotoxicity in rats was evaluated. Results showed that ACR could decrease cell viability in PC12 cells, which was suppressed by administration of rutin. Also, rutin significantly inhibited behavioural index changes and body weight loss caused by ACR administration in rats. Also, the MDA levels significantly reduced in all groups of rutin.

ACR is known to induce apoptosis in time- and dose-dependent manners in neurons and astrocytes and significantly reduce the proliferation of neural progenitor cells and, in high concentrations [31]. In our study, ACR (5.46 mM) decreased cell viability in PC12 cells and pretreatment with rutin (0.5, 1, 1.5, 2.5, 5, 10, 20 and 40 μM/ml) increased the cell viability. According to previous studies, reactive oxygen species (ROS) have important role in the toxicity of ACR [31,32].

Antioxidant effects of rutin have been established in several studies [23,33]. Rutin and catechol-compounds are electron donors, and antioxidant activities of rutin and catechol and its derivatives indicate that they are potential antioxidants [34,35]. It seems that the protective effect of rutin in this model may be at least, in part, due to suppression of ROS generation because rutin has exhibited antioxidant effect in different studies [36].

ACR monomer is a potent neurotoxin and capable of inducing CNS and PNS damages in humans and animals. ACR induces ataxia, skeletal muscle weakness and weight loss in both occupationally exposed humans and experimental animal models [26,37]. Our results showed that treatment of animals with ACR (50 mg/kg, i.p.) for 11 days decreased body weight and caused gait abnormalities, and at the end of 14 days, ACR-exposed animals displayed severe abnormal gait (scores 4), but treatment of animals with rutin prevented the decrease in body weight and reduced abnormal gait.

In recent decades, there are several studies that have shown the neuroprotective effects of rutin [38-40]. Rutin has neuroprotective effect in brain ischemia and its administration attenuates ischemic neural apoptosis by inhibition of neurological deficit, lipid peroxidation, p53 expression and increase in endogenous antioxidant defence enzymes [41]. It was shown that the 4-oxo group and the 2,3 double bond in the C ring are common in rutin and that it may be related by its neuroprotective action [42]. Rutin has beneficial effects on hypoxic, glutamate and oxidative stress at concentrations as low as 1 nM on retinal ganglion cell. It appears that the sugar side chain of flavonoids may be important for neuroprotective activities [43].

The neuroprotective effect of vitamin E is related to its antioxidant activity [44,45]. For this reason, vitamin E was used was as a positive control in protection against ACR- induced neurotoxicity. As our results, vitamin E (200 mg/kg) increased the body weight compared to rutin as a preventive agent in rats.

It seems that the effect of vitamin E in the presence of ACR after 3 days treatment in inhibition of gait abnormalities is more than rutin (200 mg/kg). However, the protective and therapeutic effects of rutin (200 mg/kg) on lipid peroxidation following ACR administration are same as vitamin E (200 mg/kg).

It is possible that rutin by inhibiting neurological deficit could improve behavioral index of animals. Moreover, it was shown that rutin might be effective in treatment of tardive dyskinesia, an extrapyramidal movement disorder, through communicating the imbalance of dopaminergic transmission [46]. Pretreatment of rutin (100 and 200 mg/kg/day) for long-term caused behavioural and neurochemical changes in aged WAG male rats. It is possible that rutin may exert this effect by affecting brain dopaminergic and adrenergic systems [47]. Thus, there is another possibility that rutin could improve behavioral index of animals via modulating dopaminergic and adrenergic transmissions.

The results of the current study suggested that protective effect of rutin against ACR cytotoxicity in PC12 cells and rats was due to inhibition of ROS production in PC12 cells exposed to ACR or in the rat nervous system under ACR treatment.

## Competing interests

The authors declare that they have no competing interests.

## Authors’ contribution

HH, designed the study, conducted, supervised experiment and prepared manuscript. SAF and VMS, conducted experiment and prepared manuscript. MNA, prepared manuscript. All authors read and approve the final manuscript.
